# Coronary artery ectasia presenting as acute coronary syndrome and misinterpreted as coronary artery perforation: Case report

**DOI:** 10.1097/MD.0000000000042330

**Published:** 2025-05-02

**Authors:** Hongki Jeon, Jin-Man Cho, In-Ho Yang, Narae Kim, Chang-Bum Park

**Affiliations:** a Department of Internal Medicine, Division of Cardiology, Kyung Hee University, Kyung Hee University Hospital at Gangdong, Seoul, Republic of Korea.

**Keywords:** acute coronary syndrome, case report, Coronary artery ectasia, coronary artery perforation, percutaneous coronary intervention, thrombectomy

## Abstract

**Rationale:**

Coronary artery ectasia (CAE), characterized by diffuse dilation, can be associated with total thrombotic occlusion, leading to acute coronary syndrome. In such cases, distal vessel morphology can be highly unpredictable, potentially causing confusion during percutaneous coronary intervention (PCI).

**Patient concerns:**

A 47-year-old man presented with sudden chest pain. Acute coronary syndrome was suspected based on symptom and elevated troponin I levels.

**Diagnoses:**

Coronary angiography revealed diffuse CAE and total occlusion of mid-left circumflex artery.

**Interventions:**

Due to the large thrombus, aspiration thrombectomy, intracoronary abciximab, and repeated balloon angioplasty were performed. After these procedures, there was absence of flow beyond the lesion, and huge extravasation around the vessel, resembling a coronary artery perforation. Considering various factors, we concluded it was not a perforation and subsequently performed intravascular ultrasound-guided PCI on the ectatic culprit vessel. After successful PCI, he was discharged on aspirin and clopidogrel. Due to heartburn, dual antiplatelet therapy was de-escalated to clopidogrel monotherapy after 6 months.

**Outcomes:**

During the follow-up, he remained stable, and a 9-month coronary angiography confirmed patent stent without lesion progression.

**Lessons:**

Stagnant flow in dilated vessels can cause local dye deposition, which may resemble procedure-induced perforation or dissection, necessitating heightened caution during PCI. Intravascular ultrasound is valuable for accurate assessment of lesions in CAE. Thrombectomy and glycoprotein IIb/IIIa inhibitors would be considered to manage high thrombus burden. Due to its diverse clinical presentations, CAE requires an individualized strategy, and can also be treated with simple PCI followed by dual antiplatelet therapy.

## 1. Introduction

Coronary artery ectasia (CAE), characterized by diffuse dilation, can sometimes be associated with total thrombotic occlusion, resulting in acute coronary syndrome (ACS).^[1]^ In such cases, distal vessel morphology can be highly unpredictable, posing challenges during interventional procedures. Clear guidelines or therapeutic strategies for such situations are currently lacking. In this report, we present a case of CAE presenting as ACS. It was initially misinterpreted as a procedure-induced coronary artery perforation, and for this reason, we aim to share our valuable experience with clinicians.

## 2. Case report

A 47-year-old man presented with sudden-onset chest pain and a history of hypertension and dyslipidemia. His blood pressure was 150/110 mm Hg, pulse rate was 68 bpm, with regular heart beat and unremarkable findings on examination. Chest X-ray showed no infiltrates or edema, and electrocardiogram revealed T-wave inversion in lead III without ST changes. The initial cardiac marker was normal, but follow-up testing after 1 hour showed an increase in high-sensitivity troponin I (5.2–19.4 pg/mL). Echocardiography revealed normal wall motion. However, persistent chest pain unresponsive to medications led to a diagnosis of non-ST elevation myocardial infarction, prompting coronary angiography (CAG).

**Figure 1.**
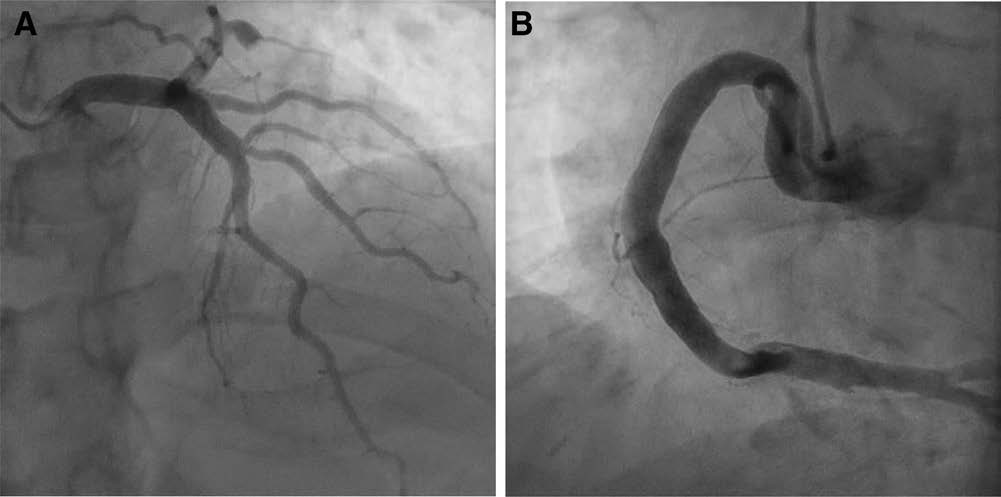
Coronary angiography. (A) Left coronary artery. (B) Right coronary artery.

**Figure 2.**
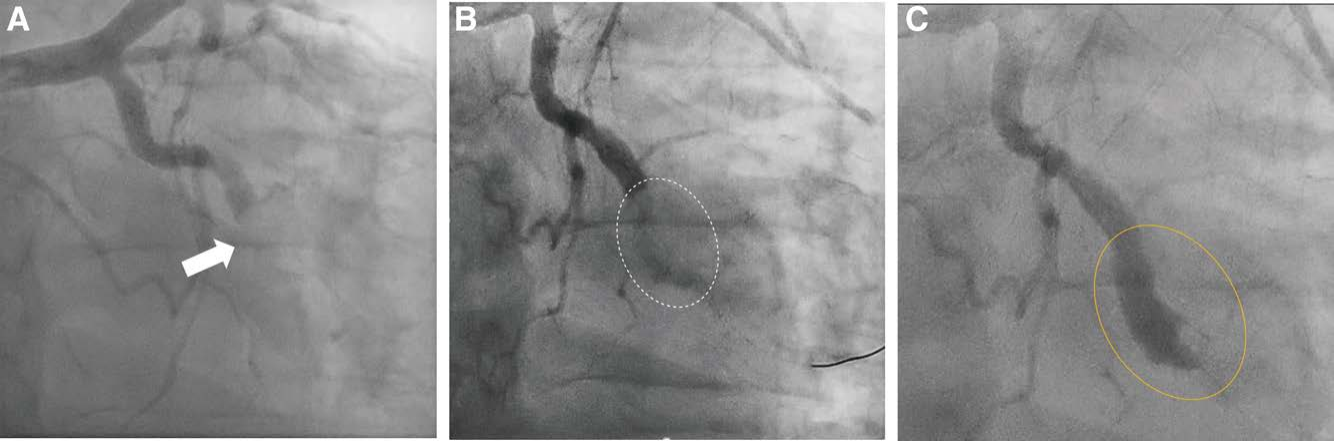
Coronary angiography. (A) Total occlusion of the mid-left circumflex artery (white arrow). (B) Significantly dilated vessel silhouette without flow beyond the lesion, resembling a coronary artery perforation (white dashed line). (C) The hidden ectatic vessel with large thrombus (orange solid line).

CAG revealed diffuse CAE (Fig. 1) and total occlusion of the mid-left circumflex artery (Fig. 2A), leading to percutaneous coronary intervention (PCI). The lesion was easily crossed with a wire, and a 2.5 mm semi-compliant balloon was used for angioplasty. However, this angioplasty failed to resolve the large thrombus and instead promoted distal embolization, worsening the flow.

To address this problem, aspiration thrombectomy, intracoronary abciximab, and repeated balloon angioplasty were performed. After these procedures, there was absence of flow beyond the lesion, and huge extravasation was observed around the vessel, resembling a coronary artery perforation (Fig 2B and Video S1, Supplemental Digital Content, https://links.lww.com/MD/O784).

**Figure 3.**
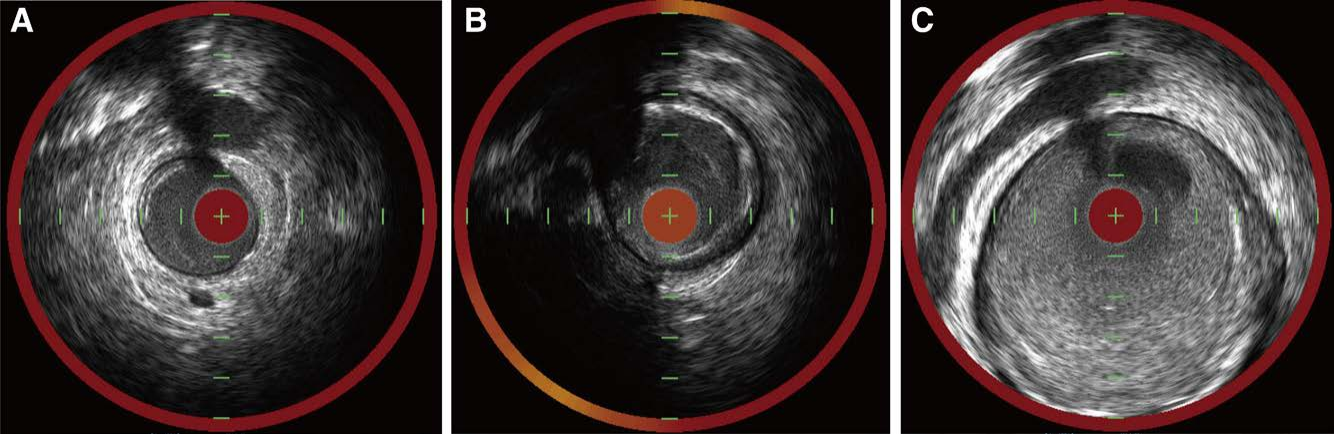
Intravascular ultrasound (IVUS) imaging. (A) Distal reference vessel. (B) Distal culprit ectasia. (C) Proximal ectasia.

The patient’s vital signs remained stable, and no changes in the cardiac silhouette were noted on the cine image, suggesting the absence of tamponade. Considering that balloon angioplasty was not performed with excessive force, our team determined procedure-induced perforation could be reasonably ruled out. Therefore, the next angiogram was obtained, revealing a hidden ectatic vessel—more dilated than the proximal ectasia—filled with a large thrombus, located just distal to the occlusion site (Fig. 2C). Intravascular ultrasound (IVUS) confirmed the size of ectasia, which was significantly larger than seen on angiography, showing a tapering shape distally with a very high thrombus burden (external elastic membrane [EEM] of distal reference vessel: diameter 3.0 mm, EEM of distal ectatic culprit vessel: diameter 4.4 mm, EEM of proximal largest ectatic vessel: diameter > 7.2 mm) (Fig. 3).

**Figure 4.**
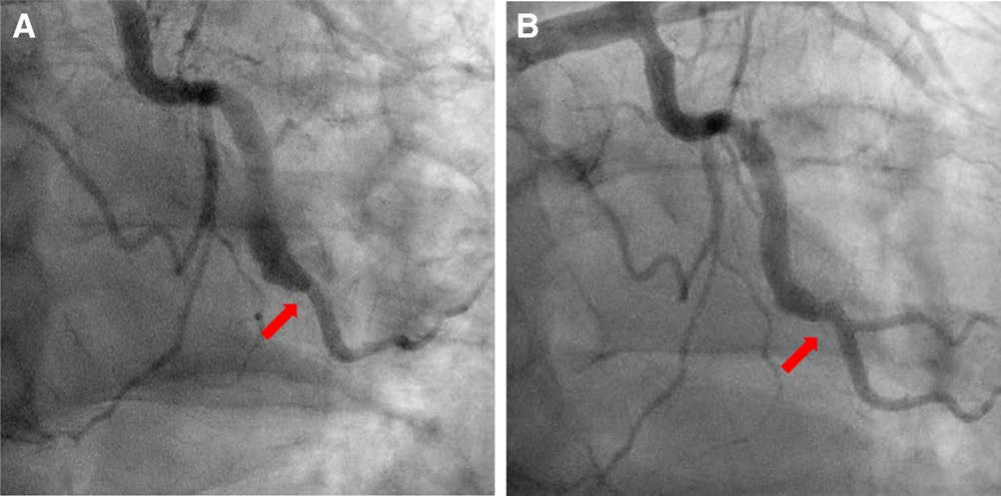
Coronary angiography and stent position (red arrow). (A) Final angiogram after stent deployment. (B) Nine-month follow-up angiography.

The proximal vessel was too large to cover, but the distal landing zone was straightforward without any lesion, so the stent was matched to the distal reference vessel. An everolimus-eluting stent (2.5–16 mm) was deployed at 2.78 mm under high pressure, restoring distal flow (Fig. 4A). The patient’s chest pain improved, and he was discharged on aspirin, clopidogrel, statin, and diltiazem. He remained stable during outpatient follow-up for 6 months but discontinued aspirin at the 6-month due to heartburn. At 9 months, a follow-up CAG showed patent stent and no progression of other lesions, as well as recovery of the distal branch vessel (Fig. 4B).

## 3. Discussion

Aneurysmal dilation of the coronary artery is observed in up to 0.3% to 5.3% of patients.^[2]^ Coronary artery aneurysm is defined as focal dilation, whereas CAE refers to diffuse dilation of at least 1.5 times the diameter of the adjacent normal segment.^[3]^ However, even with diffuse ectasia, the extent of dilation within a single coronary artery can vary, which can sometimes resemble aneurysmal changes. As seen in our case, this variability, combined with total thrombotic occlusion, can create unpredictable distal vessel morphology, posing significant challenges.

Atherosclerosis is considered the most common cause of CAE, accounting for up to 50% of adult cases.^[2]^ Although the precise pathogenesis of CAE formation remains unclear, it is thought to result from exaggerated positive vascular remodeling in response to atherosclerotic plaque growth.^[4]^ In patients with CAE, concomitant atherosclerotic disease can lead to ACS through local thrombosis. Thrombotic occlusion often occurs in the most dilated segments, where stagnant flow and abrupt changes in flow dynamics are common.^[5,6]^ This stagnant flow, which is closely associated with severity of ectasia, can also cause local dye deposition in the dilated segments, potentially mimicking perforation or dissection.^[1,7]^

Clinicians face considerable challenges when managing CAE due to its diverse clinical presentations, and limited large-scale data on treatment strategies.^[8]^ Various approaches, including medical treatment, surgical intervention, or percutaneous intervention, can be considered, requiring an individualized strategy. As in our case, if the vessel morphology is closer to diffuse ectasia rather than a focal aneurysm, PCI may be a valuable treatment option in the setting of ACS. In addition to bizarre forms of dye deposition, which could be mistaken for perforation, certain technical challenges can arise during PCI.

The first challenge is assessing the appropriate sizing and landing zone. A partially thrombosed aneurysm can result in angiographic underestimation, making IVUS essential for accurately assessing vessel diameter. Additionally, IVUS is helpful for distinguishing true aneurysms from pseudoaneurysms, dissection, or perforation, as seen in this case. It is also useful for evaluating stent malapposition, but caution is needed when performing post-stent IVUS due to the risk of stent dislodgement.^[8]^ Due to the significantly large vessel size, optical coherence tomography is less useful for evaluating CAE compared to IVUS. The second challenge is managing the high thrombus burden, which raises the risk of no-reflow. Several reports have noted higher rates of no-reflow and increased use of thrombus aspiration, in these patients.^[9,10]^ Additionally, the other report has documented the frequent use of glycoprotein IIb/IIIa inhibitors in patients with CAE treated with primary PCI.^[11]^ To prevent no-reflow and restore distal flow effectively, thrombectomy (either aspiration or mechanical) and administration of glycoprotein IIb/IIIa inhibitors would be considered before the definitive intervention.^[12,13]^

There is no clear consensus on the appropriate antithrombotic therapy for CAE patients treated with PCI. Traditionally, CAE has been associated with high thrombotic risk, and anticoagulation for primary prevention has been suggested. However, some reports of similar adverse event rates in patients with and without CAE have sparked debate over the necessity of anticoagulation.^[14,15]^ Since dual antiplatelet therapy (DAPT) is generally recommended for PCI patients and triple therapy (DAPT with anticoagulation) significantly increases bleeding risk, DAPT is considered safer. Although prolonged DAPT is sometimes recommended in patients with CAE, it must be tailored to each individual patient.^[8]^ In this case, the patient was switched to clopidogrel monotherapy after 6 months of DAPT due to gastrointestinal discomfort, and a 9-month follow-up CAG confirmed the absence of adverse coronary events. While longer-term monitoring is needed, our case report suggests that simple de-escalation strategies may provide sufficient treatment for patients with CAE.

## 4. Conclusion

Stagnant flow in dilated vessels can lead to local dye deposition, mimicking coronary perforation or dissection, so caution is necessary during PCI. Sharing this experience offers clinicians valuable insights to navigate similar situations with confidence.

## Acknowledgments

The authors appreciate the support of the catheterization laboratory staff of Kyung Hee University Hospital at Gangdong.

## Author contributions

**Conceptualization:** Hongki Jeon, Chang-Bum Park.

**Supervision:** Jin-Man Cho, Chang-Bum Park.

**Visualization:** In-Ho Yang, Narae Kim.

**Writing – original draft:** Hongki Jeon.

**Writing – review & editing:** Jin-Man Cho, Chang-Bum Park.

## Supplementary Material


